# Quantifying orthogonal barcodes for sequence census assays

**DOI:** 10.1093/bioadv/vbad181

**Published:** 2023-12-20

**Authors:** A Sina Booeshaghi, Kyung Hoi (Joseph) Min, Jase Gehring, Lior Pachter

**Affiliations:** Division of Biology and Biological Engineering, California Institute of Technology, Pasadena, CA 91125, United States; Department of Computer Science and Electrical Engineering, Massachusetts Institute of Technology, Cambridge, MA 02139, United States; Arcadia Science, Berkeley, CA 94702, United States; Division of Biology and Biological Engineering, California Institute of Technology, Pasadena, CA 91125, United States; Department of Computing and Mathematical Sciences, California Institute of Technology, Pasadena, CA 91125, United States

## Abstract

**Summary:**

Barcode-based sequence census assays utilize custom or random oligonucloetide sequences to label various biological features, such as cell-surface proteins or CRISPR perturbations. These assays all rely on barcode quantification, a task that is complicated by barcode design and technical noise. We introduce a modular approach to quantifying barcodes that achieves speed and memory improvements over existing tools. We also introduce a set of quality control metrics, and accompanying tool, for validating barcode designs.

**Availability and implementation:**

https://github.com/pachterlab/kb_python, https://github.com/pachterlab/qcbc.

## 1 Introduction

Single-cell RNA sequencing assays rely on unique sequences of nucleotides (“barcodes”) to associate sequences from mRNA molecules with individual cells. This strategy has been extended to quantify orthogonal biological or technical information in what is known as “multimodal” genomics. For example, the 10× Genomics Feature Barcoding assay quantifies the expression of cell-surface proteins using targeted antibody barcodes (“Feature Barcode Overview—Software—Single Cell Gene Expression—Official 10× Genomics Support” n.d.). The ClickTag (Gehring *et al.* 2020), Multiseq (McGinnis *et al.* 2019), and Cell Hashing (Stoeckius *et al.* 2018) assays, on the other hand, use custom sample-specific barcodes to group cells enabling multiplexing and reducing batch effects. Moreover, similar approaches have been used for CRISPR screens (Dixit *et al.* 2016, Datlinger *et al.* 2017, Schraivogel *et al.* 2020), targeted perturbation assays (Gehring *et al.* 2020, Srivatsan *et al.* 2020), and recently for spatial genomics assays (Srivatsan *et al.* 2021).

The processing of data from these assays has required the development of custom tools, resulting in a proliferation of application- and assay-specific software (Roelli *et al.* 2019, Johnson *et al.* 2023). We introduce a broadly applicable framework, which we term “*kITE*” for ***k****allisto* **I**ndexing and **T**ag **E**xtraction, for uniformly quantifying orthogonal barcode-based sequence census assays that has several advantages over assay-specific tools. First, by virtue of providing a single solution to seemingly distinct problems, it serves as a unifying framework for *biology x barcode* assays and will simplify the development and deployment of novel assays. Second, the software implementation we share is portable, engineered to work across platforms, and is modular, allowing for easy adaptation and customization. It makes use of the *kallisto* and *bustools* programs that have been previously extensively validated and benchmarked for quantification of the biological sequences in *barcode x biology* assays such as single-cell and bulk RNA sequencing (Bray *et al.* 2016, Melsted *et al.* 2021). Finally, our method is faster than existing tools, and we identify corner cases in processing that have been missed in previous analyses. The examination of such cases led us to develop a tool, called *qcbc*, for assessing the quality of barcode sets, and that should prove to be a useful standalone package. It is applicable both during assay development, and during assay quality control.

## 2 Methods

**Figure 1.**
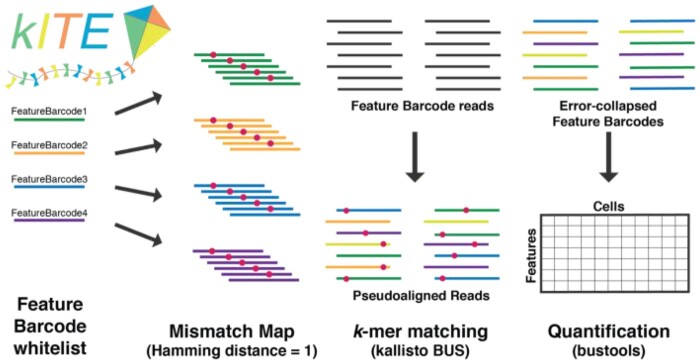
*kITE* workflow. The mismatch map is generated from the feature barcode whitelist and indexed with *kallisto*. Reads are pseudoaligned and error-collapsed with *kallisto* and *bustools* to form a cells x features matrix.

Feature Barcoding assays use short barcodes and are susceptible to ambiguous or incorrect assignment if errors are introduced during the experimental procedure. Inspired by the *CITE-seq-count* approach, *kITE* begins by generating a list of all single-base mismatches of the Feature Barcodes used in an experiment (Fig. 1). The resulting “mismatched FASTA” file is used as input for the “*kallisto* index” program with *k* set appropriately (Section 2). Finally, the *kallisto* | *bustools* pipeline is used to quantify the dataset using the “mismatch index” generated after running “*kallisto* index” on the mismatched FASTA file. In this way, *kallisto* will effectively scan the entire sequencing read for barcodes present in the mismatch index allowing for barcode pseudoalignment despite variability in barcode position. Feature barcode “error correction” occurs when barcode counts are summed across different mismatches of a given barcode (Section 2).

## 3 Results

**Figure 2.**
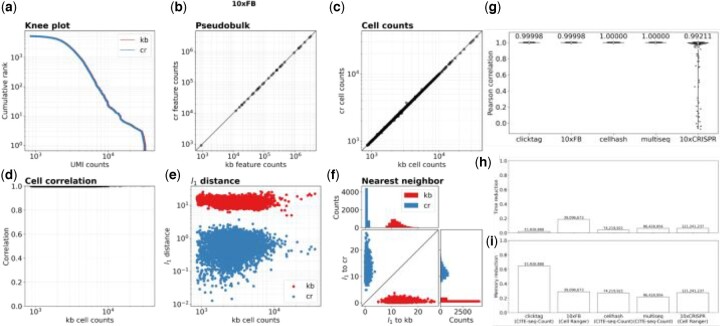
Comparison between *kallisto bustools* (kb) quantifications and Cell Ranger (cr) quantifications for the 10× Feature Barcoding assay on 5266 Peripheral blood mononuclear cells dataset targeting 32 cell surface proteins. (a) Knee plot comparing cumulative UMI counts per cell. (b) Pseudobulk comparison of cumulative UMI counts per feature barcode. (c) Cumulative UMI counts per cell. (d) Pearson correlation of the same cell between the two quantifications. (e) The*^l^*1 distance between a *kallisto bustools* cell and its Cell Ranger equivalent and the same cell and its nearest *kallisto bustools* neighbor across the total UMI counts for that cell. (f) The ^*l*^1 distance of a *kallisto bustools* cell to its Cell Ranger equivalent (*y*-axis) and to its nearest neighbor (*x*-axis) and the*^l^*1 distance of a Cell Ranger cell to it’s *kallisto bustools* equivalent (*x*-axis) and its nearest neighbor (*y*-axis). The marginal distributions show that each *kallisto bustools* cell is closest to its corresponding Cell Ranger cell and that each Cell Ranger cell is nearest to its corresponding *kallisto bustools* cell. (g) Pearson correlation for all the cells between the *kallisto bustools* qualifications and in *CITE-seq-Count* or *Cell Ranger* quantifications for each of the five datasets. (h) Runtime and (i) memory improvements for each of the five datasets across the various read depths.

To evaluate the accuracy and efficiency of *kITE* across a range of assays, we gathered data from six different assays, and compared *kITE* quantifications to those produced with assay-specific tools. We first quantified cell surface protein abundance for the 10× Genomics 5266 Peripheral blood mononuclear cells dataset, which targeted 32 cell surface proteins. We used *kallisto bustools* and a mismatch index to pseudoalign the target sequences (Appendix A.1.1) and found high concordance between our method and *Cell Ranger* across varying read depths, with an average Pearson correlation of 0.99 (Fig. 2a–f). *kITE* was five times faster than *Cell Ranger* and required 3.5 times less RAM (Appendix A.1.2).

Next, we tested how accurately we could quantify and demultiplex pooled samples tagged with sample-specific barcodes. We processed data from 3795 methanol-fixed mouse neural stem cells from four multiplexed samples generated with the ClickTag assay (Supplementary Fig. S1), 9551 primary patient-derived xenograft (PDX) samples from nine multiplexed samples generated with the Multiseq assay (Supplementary Fig. S2) and 10 807 PBMCs from eight multiplexed samples generated with the Cell Hashing assay (Supplementary Fig. S3). We found high concordance with an average Pearson correlation of 0.99, for all three datasets, between *kITE* and *CITE-seq-Count* (Roelli *et al.* 2019), a tool for counting antibody tags from a CiteSeq or Cell Hashing experiments (Fig. 2g). *kITE* was 48 times faster and required only a quarter to a fifth of the RAM (Fig. 2h and i). We observed slight discordance with two feature barcodes, BC49 and BC74, when processing the PDX samples assayed with Multiseq. These two Multiseq barcodes are short (8 bp in length), yet share long subsequences up to 7 bp in length (Supplementary Fig. S4a). The introduction of errors during sequencing can result in possible incorrect barcode assignment and loss of barcodes (Appendix A.1.3). We addressed this issue by trimming the excess sequence surrounding the 8-bp feature barcode (Appendix A.1.6). We also observed a discordance on a subset of cells in the 10×CRISPR assay between kITE quantifications and Cell Ranger. Investigation of the unmapped Cell Ranger barcodes indicates that Cell Ranger failed to map reads with a clear guide-of-origin. For example, Cell Ranger failed to map 43 098 reads (8312 UMIs) for the cell CTGAGCGCATAAGATG with a guide RNA that exactly matches that for DPP4-1 (Section 2).

To demonstrate the flexibility of our approach we also processed 2799 A549 lung carcinoma cells expressing nuclease-dead Cas9–Krüppel-associated box (dCas9–KRAB) assayed with the 10× Genomics CRISPR screen kit, and transduced with 90 sgRNAs targeting 45 different genes. We achieve high concordance against *Cell Ranger* with an average Pearson correlation of 0.99 across all cells for nearly sgRNA’s, with two sgRNAs in the 10× CRISPR screen dataset, EZR-1 and PPIB-2, differing substantially (Supplementary Fig. S5). Both of these two sgRNA’s share a subsequence of 16 bp with EZR-2 and PPIB-1 respectively. The latter two sequences begin with GCG and GGA, both of which are a single hamming distance away from the three bases that precede the target sgRNA in the protospacer sequence (Supplementary Fig. S4b). Therefore, a single mutation two bases away from the start of the EZR-1 sgRNA sequence could result in an ambiguous read. To resolve this, we trimmed the protospacer sequence off of the reads (Appendix A.1.8).

We also processed 10 860 K562 cells expressing dCas9–KRAB from TAP-Seq transduced with 86 sgRNAs targeting 14 promoters or enhancers and 30 control sgRNAs to compare assignment of sgRNAs to individual cells. We found 97.8% of all 10 860 barcodes are fully concordant with TAP-Seq assignments, 0.53% of cells are partially concordant, and 1.6% of cells are fully discordant. (Appendix A.1.10). The discrepancies are likely the result of differences between mapping procedures producing differences in counts near the threshold (8 UMI counts) resulting in differential cell assignment for cells with low UMI counts (Supplementary Fig. S6).

### 3.1 Read trimming

Sequencing libraries often contain adapter or spurious sequences that flank the barcode and the presence of these shared sequences can make mapping challenging. Sequencing reads are often trimmed, with tools such as *trimmomatic* (Bolger *et al.* 2014), to avoid mapping noninformative regions that flank a barcode, a task that is crucial when barcodes share subsequences at their ends. Trimming helps to avoid longer sequence clashes that contain both the shared barcode subsequence and the shared nonbarcode sequence. The 10×CRISPR assay, e.g. contains two barcodes which share a 16-bp subsequence (the ends of which are one hamming distance from a subsequence of the protospacer sequence that flank the barcode) (Supplementary Fig. S4). Similarly, the Multiseq assay produces sequencing reads where the barcodes are flanked by poly-A stretches and two barcodes share a 7-bp subsequence that contains the end of the barcode.

### 3.2 Barcode design validation

**Figure 3.**
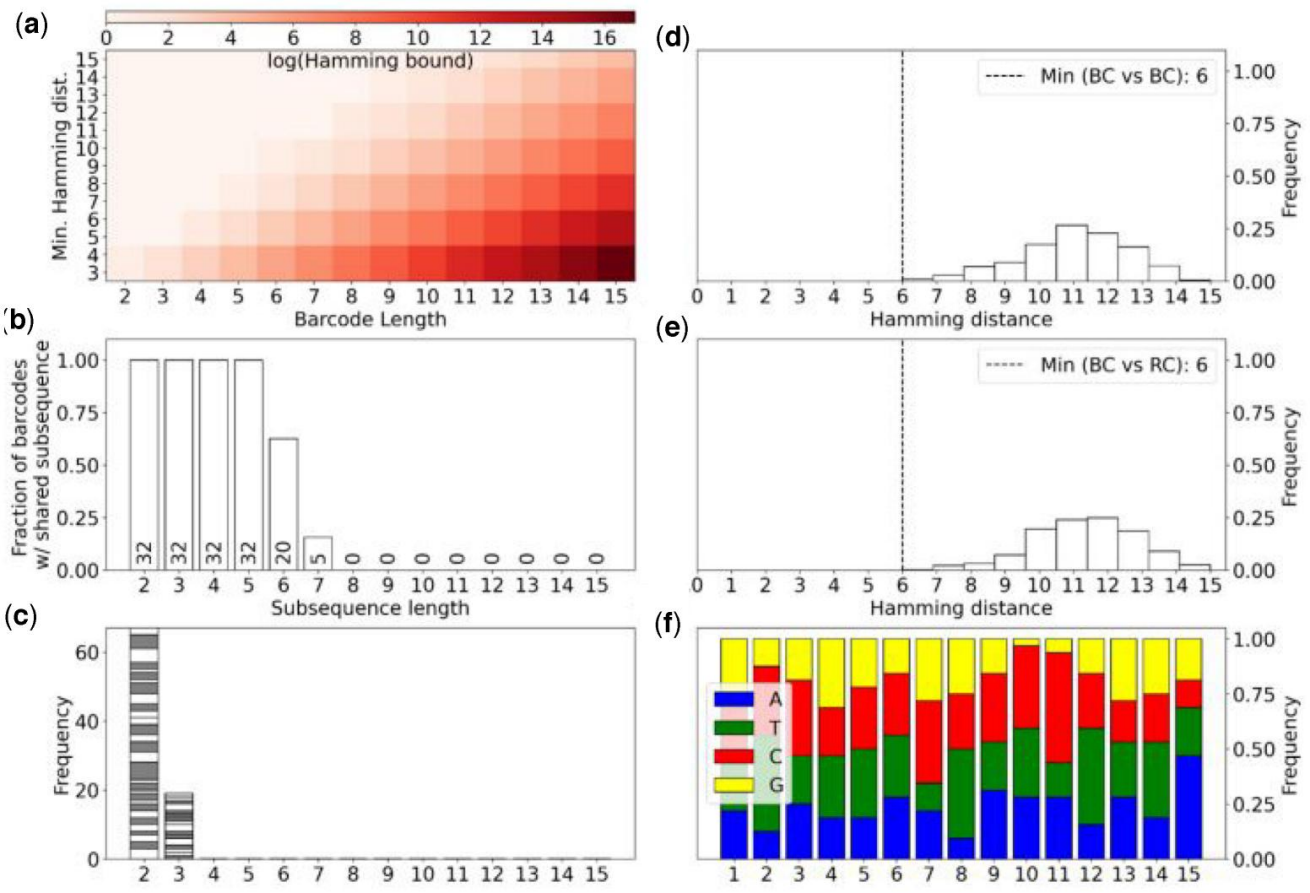
Evaluation of barcode designs. (a) Potential library diversity for 4-letter DNA barcodes of various lengths subject to a minimum pairwise Hamming distance constraint. (b) Number of ambiguous barcodes for varying subsequence length. (c) The distribution of the number of homopolymer stretches of a given length. The different color bands indicate a unique barcode and the size of the band indicates the number of homopolymers of that length found in that barcode. (d) Distribution of pairwise hamming distances between barcodes. The minimum pairwise Hamming distance is marked with a dashed line. (e) Distribution of pairwise hamming distances between barcodes and their reverse complement. The minimum pairwise Hamming distance is marked with a dashed line. (f) The per base nucleotide content across all barcodes.

Orthogonal barcode sequences designed for quantifying multimodal data, demultiplexing samples, or performing large-scale perturbation experiments can clash if the sequences are not sufficiently unique. To allow researchers to assess the extent of these issues in datasets they are analyzing, or for assays they are developing, we developed a quality control tool, *qcbc*, and a simulation framework to assess the quality of a set of barcodes. Our tool is complementary to the simulation framework proposed in (Johnson *et al.* 2023) and expands on the functionality of DNABarcodes (Buschmann 2017) which produces pairwise barcode distance comparisons. *qcbc* computes multiple metrics for barcode quality control:

1. diversity of barcodes (subject to distance constraints) (Fig. 3a),
2. number of barcodes that share a subsequence of a given length (Fig. 3b),
3. distribution of Hamming distances between pairwise barcodes (Fig. 3c and d),
4. distribution of homopolymers of a given length (Fig. 3e), and
5. per position and barcode nucleotide distribution (Fig. 3f, Supplementary Fig. S7).

With *qcbc* we are able to identify the 16-bp shared subsequence with EZR-2 and PPIB-1 sgRNAs in the 10×CRISPR that result in improper quantification. *qcbc* is also able to identify the 7-bp shared subsequence between and BC74 in the Multiseq assay.

These metrics are important for understanding the impact that barcode error correction has on rescuing sequencing reads. A large diversity of unique barcodes with shorter shared subsequences and larger pairwise distances between barcodes make it easier to disambiguate sequencing reads with pseudoalignment. Additionally, long homopolymer runs and low nucleotide content diversity could contribute to sequencing errors (“Illumina: What Is Nucleotide Diversity and Why Is It Important?” 2016, Sina Booeshaghi and Pachter 2022).

To evaluate the impact that base errors have on error correction, we developed a simulation framework that reports the fraction of lost barcodes for a given error rate in the observed sequences (Supplementary Fig. S8) (Appendix A.1.13). Short barcodes, such as those in the Multiseq assay, can yield insufficient barcode diversity that results in a greater fraction of barcodes that cannot be unambiguously assigned (Supplementary Fig. S9). Barcodes with small minimum-pairwise/edit distance (Fig. 3d and e) and long shared subsequences (Fig. 3b) such as those in the Mulitseq and 10×CRISPR assay can result in more clashes (Appendix A.1.12).

## 4 Discussion

We have demonstrated a broadly applicable approach to align, error-correct, group, and count sequences from orthogonal barcodes to quantify multimodal data such as cell surface protein abundance, to demultiplex samples, and to perform large-scale perturbation experiments. Our approach is fast, memory efficient, and extensible: orthogonal barcode quantifications can be obtained for numerous assays with variable sequence structure such the recently developed spatial RNA-seq assays (Srivatsan *et al.* 2021) and massively parallel reporter assays (Gordon *et al.* 2020). This allows future assay developers to avoid producing, validating, and maintaining software implementing *ad hoc* solutions. Moreover, some current methods are slow, have high memory requirements, and are specifically tailored for specific assays, which can result in batch effects when performing integrative analysis. Methods such as *CITE-seq-Count* offer extensibility features for different barcode strategies, but are slow and memory-intensive. The speed and efficiency of our approach enables reproducible single-cell analysis in the cloud with tools such as Google Colab. We anticipate that our framework for quantifying orthogonal barcoding assays will be useful as multimodal assays proliferate (Packer and Trapnell 2018), and that our quality control tool will help assay developers avoid pitfalls in barcode design and help practitioners quality control orthogonal barcode assays before generating data. Finally, while our approach is currently engineered to correct mismatch errors, robustness to other types of sequencing errors are possible via other methods that could readily be incorporated in our modular framework (Zorita *et al.* 2015, Sina Booeshaghi *et al.* 2020, Johnson *et al.* 2023).

## Supplementary Material

vbad181_Supplementary_Data

## Appendix

### A.1 Methods

All code, parameter options, and data can be found in the paper’s GitHub repository: https://github.com/pachterlab/BMGP_2020. *kite* is implemented in *kb_python* and can be found here: https://github.com/pachterlab/kb_python. The *qcbc* barcode validator can be found: https://github.com/pachterlab/qcbc.

#### A.1.1 Mismatch index, pseudoalignment, and counting

To quantify against a known set of target sequences we first build an alignment index containing all Hamming distance = 1 variants of each target. Restricting the alignment index to Hamming distance = 1 variants allows for recovering more reads while keeping the index size small (Supplementary Fig. S10). The hamming-1 index for the Clicktag assay is 72.8 KB and the hamming-0 index is 2.1 KB. Note that we generate the kallisto index (de Bruijn Graph) for all orthogonal barcodes, including all hamming 1 distance variants of each barcode (one base-pair mutation). We do not include entries that collide as they will produce ambiguities in mapping. Additionally, we set the k-mer size to be the length of the orthogonal barcode, if the barcode length is odd, or one minus the length of the orthogonal barcode, if the barcode length is even, since the k-mer length must be odd (Bray *et al.* 2016).

Targeted sequencing reads are then pseudoaligned to the custom index using *kallisto* to generate a Barcode, UMI, Set (BUS) file (Melsted *et al.* 2019). UMI counts are then aggregated per cell and quantified per feature barcode using *bustools* to generate a target count matrix.

#### A.1.2 Speed and memory comparison

Preprocessing steps were benchmarked with GNU time -v option, and the elapsed (wall clock) time and maximum resident set size (kbytes) were used to perform the time and memory comparisons. All code was benchmarked on Ubuntu 18.04.5 LTS (GNU/Linux 4.15.0–136-generic x86_64) with Intel(R) Xeon(R) CPU E5-2697 v2 @ 2.70 GHz processors.

#### A.1.3 Barcode design evaluation

qcbc allows users to evaluate potential orthogonal barcode designs. The tool reports multiple metrics that may help users understand potential error modes. Each of the metrics is explained below along what various values mean:

1. diversity of unique barcodes (subject to distance constraints) (Fig. 3a),
  - If the diversity of barcodes is high, relative to the maximum possible number of barcodes (constrained by a given pairwise distance), then the design space of barcodes was used more effectively.
  - Opting for a shorter barcode length at a given pairwise Hamming distance constraint may save reagents and assay complexity.
2. number of barcodes that share a subsequence of a given length (Fig. 3b),
  - If the number of barcodes that share a subsequence of a given length is high, then k-mer based mapping may yield multimapping reads. For example, if the barcode length is 16 bp and multiple barcodes share a 15-bp subsequence, then ambiguity in k-mer based mapping will arise.
  - Opting for barcodes with shorter ambiguous subsequences enable effective barcode discrimination using k-mer based mapping.
3. distribution of Hamming distances between pairwise barcodes (Fig. 3c and d)
  - If the distribution of pairwise Hamming distances between barcodes is low, then the mismatched index may yield barcodes that share a common subsequence giving rise to multimapping reads.
  - Opting for larger pairwise distances in barcode design will enable effective discrimination of barcodes at a given Hamming distance, rescuing more reads.
4. distribution of homopolymers of a given length (Fig. 3e)
  - If the distribution of homopolymers of a given length is high, then sequencing with technologies such as Ultima Genomics (Sina Booeshaghi and Pachter 2022) which uses mostly natural synthesis for sequencing, may yield errors.
  - Opting for fewer homopolymers may help alleviate this issue.
5. per position and barcode nucleotide distribution (Fig. 3f, Supplementary Fig. S7)
  - If the per position and barcode nucleotide distribution is not uniform, then sequencing errors may arise during basecalling.
  - Opting for a more uniform distribution of nucleotides may help improve base calling during sequencing with technologies such as Illumina.

#### A.1.4 10× feature barcode

FASTQ files for 5266 Peripheral blood mononuclear cells dataset targeting 32 cell surface proteins were downloaded from https://support.10xgenomics.com/single-cell-gene-expression/datasets/3.0.2/5k_pbmc_protein_v3. A mismatch index was created using the Feature Reference file and *kb* ref with the *kite* workflow. The reads were pseudoaligned and a feature count matrix was made using *kb* count with the kite: 10×FB technology option. *Cell Ranger*, the Feature Reference, and refdata-gex-GRCh38-2020-A was used to process the FASTQ files into a feature count matrix.

All preprocessing steps are runnable via a Google Colab notebook: https://github.com/pachterlab/BMGP_2020/blob/main/analysis/notebooks/10xFB/10xFB_preprocess.ipynb. All matrix comparisons are runnable via a Google Colab notebook: https://github.com/pachterlab/BMGP_2020/blob/main/analysis/notebooks/10xFB/10xFB_preprocess.ipynb

#### A.1.5 ClickTag

FASTQ files for 3795 methanol-fixed mouse neural stem cells from four multiplexed samples were downloaded from https://caltech.box.com/shared/static/zqaom7yuul7ujetqyhnd4lvf8vhzqsyg.gz. A mismatch index was created using the list of targeted barcodes file and *kb* ref with the *kite* workflow. The list of barcodes can be found here: https://github.com/pachterlab/BMGP_2020/tree/main/references/clicktag. The reads were pseudoaligned and a feature count matrix was obtained using *kb* count with the *kite* technology option. *CITE-seq-Count* and the targeted barcodes were used to process the FASTQ files into a feature count matrix.

All preprocessing steps are runnable via a Google Colab notebook: https://github.com/pachterlab/BMGP_2020/blob/main/analysis/notebooks/clicktag/clicktag_preprocess.ipynb. All matrix comparisons are runnable via a Google Colab notebook: https://github.com/sbooeshaghi/BMGP_2020/blob/main/analysis/notebooks/clicktag/clicktag_preprocess.ipynb

We generated a *kallisto* index with no mismatches by running *kb* ref with the—no-mismatches option and compared the resultant count matrix generated with *kb* count to the count matrix generated with mismatches.

#### A.1.6 Multiseq

FASTQ files for 9551 primary patient-derived xenograft (PDX) samples from nine multiplexed samples were downloaded from https://caltech.box.com/shared/static/0scoe38xvcoxfd62xnm848pimh2zr1ip.gz. A mismatch index was created using the list of targeted barcodes file and *kb* ref with the *kite* workflow. The list of barcodes can be found here: https://github.com/pachterlab/BMGP_2020/tree/main/references/multiseq. The reads were pseudoaligned and a feature count matrix was obtained using *kb* count with the *kite* technology option. *CITE-seq-Count* and the targeted barcodes were used to process the FASTQ files into a feature count matrix.

All preprocessing steps are runnable via a Google Colab notebook: https://github.com/pachterlab/BMGP_2020/blob/main/analysis/notebooks/multiseq/multiseq_preprocess.ipynb. All matrix comparisons are runnable via a Google Colab notebook: https://github.com/sbooeshaghi/BMGP_2020/blob/main/analysis/notebooks/multiseq/multiseq.ipynb

The FASTQ reads were trimmed using *seqtk* trimfq with the -e option set to the length of the read minus the 8 such that only the 8-bp barcode was retained.

#### A.1.7 Cell hashing

FASTQ files for 10807 PBMCs from eight multiplexed samples were downloaded from https://caltech.box.com/shared/static/0scoe38xvcoxfd62xnm848pimh2zr1ip.gz. A mismatch index was created using the list of targeted barcodes file and *kb* ref with the *kite* workflow. The list of barcodes can be found here: https://github.com/pachterlab/BMGP_2020/tree/main/references/multiseq. The reads were pseudoaligned and a feature count matrix was obtained using *kb* count with the *kite* technology option. *CITE-seq-Count* and the targeted barcodes were used to process the FASTQ files into a feature count matrix.

All preprocessing steps are runnable via a Google Colab notebook: https://github.com/pachterlab/BMGP_2020/blob/main/analysis/notebooks/cellhash/cellhash_preprocess.ipynb. All matrix comparisons are runnable via a Google Colab notebook: https://github.com/pachterlab/BMGP_2020/blob/main/analysis/notebooks/cellhash/cellhash.ipynb

#### A.1.8 10× CRISPR

FASTQ files for 2799 A549 lung carcinoma cells expressing nuclease-dead Cas9–Krüppel-associated box (dCas9–KRAB) from 10× Genomics CRISPR screen transduced with 90 sgRNAs targeting 45 different genes were downloaded from https://support.10xgenomics.com/single-cell-gene-expression/datasets/4.0.0/SC3_v3_NextGem_DI_CRISPR_10K. A mismatch index was created using the Feature Reference file and *kb* ref with the *kite* workflow. The reads were pseudoaligned and a feature count matrix was obtained using *kb* count with the kite: 10×FB technology option. *Cell Ranger*, the Feature Reference, and refdata-gex-GRCh38-2020-A was used to process the FASTQ files into a feature count matrix.

All preprocessing steps are runnable via a Google Colab notebook: https://github.com/pachterlab/BMGP_2020/blob/main/analysis/notebooks/10xCRISPR/10xCRISPR_preprocess.ipynb. All matrix comparisons are runnable via a Google Colab notebook: https://github.com/pachterlab/BMGP_2020/blob/main/analysis/notebooks/10xCRISPR/10xCRISPR.ipynb

The FASTQ reads were trimmed using *seqtk* trimfq with the -e option set to the length of the read minus the 19 plus overhang such that only the 19-bp barcode plus the set overhang was retained.

#### A.1.9 10× CRISPR barcode discordance

We took the kITE and Cell Ranger count matrices and looked for the cell barcode that was the most discordance in the sum of counts between the two quantifications (CTGAGCGCATAAGATG). We then looked for the guide RNA that exhibited the most discordant counts (DPP4-1, GCGCAGGCAGAAGTCACCG). We filtered the Cell Ranger BAM file for reads that were unaligned and then further filtered for the specific cell barcode that exhibited the most discordance. Then we grep’ed for the sequence of the specific guide RNA, that was the most discordant in the cell of interest, counted the number of reads and UMIs for which there was a direct match.

#### A.1.10 TAP-Seq

FASTQ files for 10860 K562 cells expressing dCas9–KRAB from TAP-Seq transduced with 86 sgRNAs targeting 14 promoters or enhancers and 30 control sgRNAs were downloaded from https://www.ncbi.nlm.nih.gov/sra/?term=SRR9916613. A mismatch index was created using the list of targeted barcodes file and *kb* ref with the *kite* workflow. The list of barcodes can be found here: https://github.com/pachterlab/BMGP_2020/tree/main/references/tapseq. The reads were pseudoaligned and a feature count matrix was obtained using *kb* count with the standard technology option. Perturbation status for each cell was downloaded from https://www.ncbi.nlm.nih.gov/geo/query/acc.cgi?acc=GSM4012688 using *ffq* GSM4012688 (Gálvez-Merchán *et al.* 2023).

All preprocessing steps are runnable via a Google Colab notebook: https://github.com/pachterlab/BMGP_2020/blob/main/analysis/notebooks/tapseq/tapseq_preprocess.ipynb. All matrix comparisons are runnable via a Google Colab notebook: https://github.com/pachterlab/BMGP_2020/blob/main/analysis/notebooks/tapseq/tapseq.ipynb

#### A.1.11 Datasets and code

The following table lists all datasets and code used to evaluate kITE.

**Table:**

Assay	ncells	nfeatures	barcodes	fastqs	preprocess	analysis
10×FB	5266	32	link	Link	link	link
Clicktag	3795	4	link	Link	link	link
Multiseq	9551	9	link	Link	link	link
Cell Hashing	10 807	8	link	Link	link	link
10× CRISPR	2799	90	link	Link	link	link
TAP-Seq	10 860	86	link	Link	link	link

#### A.1.12 Barcode validation

For a given list of barcodes we report the maximum number of barcodes that could be made for the given barcode length, the number of barcodes that share a subsequence of length k, and the pairwise hamming distance between all pairs of barcodes. A Google Colab notebook reproducing the results of this simulation can be found here: https://github.com/pachterlab/BMGP_2020/blob/main/analysis/notebooks/barcode_validator.ipynb

The *qcbc* tool for barcode validation can be found here: https://github.com/pachterlab/qcbc

#### A.1.13 Barcode simulation

We assumed a per-base error rate that is constant for every base in a barcode and simulated barcode mutants from a set of given barcodes. Then, for multiple error rates, we estimated the number of barcode mutants that are a given hamming distance from the correct barcode that became ambiguous after introducing sequencing errors. A Google Colab notebook reproducing the results of this simulation can be found here: https://github.com/pachterlab/BMGP_2020/blob/main/analysis/notebooks/barcode_sim.ipynb

### A.2 Software versions

Anndata 0.6.22.post1

bustools 0.41.0

Cell Ranger 6.0.1

CITE-seq-Count 1.4.4

awk (GNU awk) 4.1.4

grep (GNU grep) 3.1

kallisto 0.48.0

kb_python 0.26.3

Matplotlib 3.0.3

Numpy 1.18.1

Pandas 0.25.3

Scipy 1.4.1

sed (GNU sed) 4.4

seqtk 1.2-r94

sklearn 0.22.1

tar (GNU tar) 1.29
